# *N*-(2-Methoxy­ethyl)phthalimide

**DOI:** 10.1107/S1600536808013548

**Published:** 2008-05-10

**Authors:** Yoke Leng Sim, Azhar Ariffin, Seik Weng Ng

**Affiliations:** a Department of Chemistry, University of Malaya, 50603 Kuala Lumpur, Malaysia

## Abstract

The title mol­ecule, C_11_H_11_NO_3_, lies on a crystallographic mirror plane which bis­ects the plane of the phthalimide unit and contains the C and O atoms of the 2-methoxy­ethyl group.

## Related literature

For medicinal properties of the title compound, see: Chapman *et al.* (1989 ▶); Hall *et al.* (1994 ▶). For a kinetic study of the reaction that yields the title compound, see: Khan (1994 ▶).
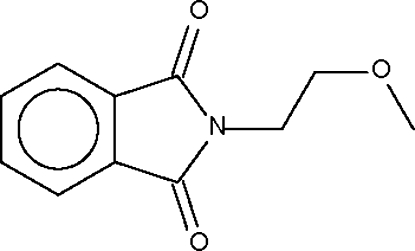


## Experimental

### 

#### Crystal data

C_11_H_11_NO_3_
*M* *_r_* = 205.21
Orthorhombic, 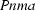

*a* = 7.0514 (2) Å
*b* = 9.3852 (2) Å
*c* = 14.6024 (4) Å
*V* = 966.37 (4) Å^3^
*Z* = 4
Mo *K*α radiation
μ = 0.10 mm^−1^
*T* = 100 (2) K
0.30 × 0.20 × 0.10 mm

#### Data collection

Bruker SMART APEX diffractometer
Absorption correction: none
7349 measured reflections
1164 independent reflections
986 reflections with *I* > 2σ(*I*)
*R*_int_ = 0.039

#### Refinement

*R*[*F* ^2^ > 2σ(*F* ^2^)] = 0.059
*wR*(*F* ^2^) = 0.206
*S* = 1.11
1164 reflections
77 parameters
H-atom parameters constrained
Δρ_max_ = 0.50 e Å^−3^
Δρ_min_ = −0.50 e Å^−3^

### 

Data collection: *APEX2* (Bruker, 2007 ▶); cell refinement: *SAINT* (Bruker, 2007 ▶); data reduction: *SAINT*; program(s) used to solve structure: *SHELXS97* (Sheldrick, 2008 ▶); program(s) used to refine structure: *SHELXL97* (Sheldrick, 2008 ▶); molecular graphics: *X-SEED* (Barbour, 2001 ▶); software used to prepare material for publication: *publCIF* (Westrip, 2008 ▶).

## Supplementary Material

Crystal structure: contains datablocks global, I. DOI: 10.1107/S1600536808013548/lh2624sup1.cif

Structure factors: contains datablocks I. DOI: 10.1107/S1600536808013548/lh2624Isup2.hkl

Additional supplementary materials:  crystallographic information; 3D view; checkCIF report

## Figures and Tables

**Fig. 1.**
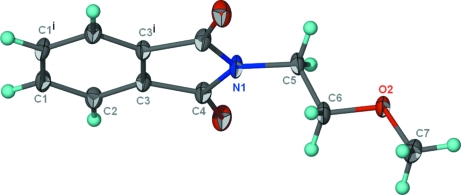
Thermal ellipsoid plot (Barbour, 2001) of C11H11NO3 at the 70% probability level. Hydrogen atoms are drawn as spheres of arbitrary radiius. Symmetry transformation (i): x, 1/2 – y, z.

## supplementary crystallographic information

### Comment

The title compound was previously reported in a kinetic study (Khan, 1994). We intend to carry out studies on the medicinal properties of the compound; some such properties have been reported (Chapman *et al.*, 1989; Hall *et al.*, 1994). The molecule of *N*-(2-methoxyethyl)phthalimide lies on a mirror plane that relates one half of the phthalamido portion of the molecule to the other; the 2-methoxyethyl substituent lies on the mirror plane itself (Fig. 1).

### Experimental

Phthalic anhydride (2.59 g, 17.5 mmol) and 2-methoxyethylamine (1.50 ml, 17.5 mmol) were dissolved in acetic acid (25 ml). The mixture was heated at 393–413 K for 4 h; the reaction was monitored by TLC. Water was added to precipitate the product, which was collected (80% yield.) Crystals were obtained upon recrystallization from water.

### Refinement

Carbon-bound H-atoms were placed in calculated positions (C—H 0.95 to 0.99 Å) and were included in the refinement in the riding model approximation, with *U*(H) set to 1.2–1.5 *U*(C).

### Crystal data

**Table:**

C_11_H_11_NO_3_	*F*_000_ = 432
*M**_r_* = 205.21	*D*_x_ = 1.410 Mg m^−^^3^
Orthorhombic, *P**n**m**a*	Mo *K*α radiation λ = 0.71073 Å
Hall symbol: -P 2ac 2n	Cell parameters from 2463 reflections
*a* = 7.0514 (2) Å	θ = 2.6–28.3º
*b* = 9.3852 (2) Å	µ = 0.10 mm^−^^1^
*c* = 14.6024 (4) Å	*T* = 100 (2) K
*V* = 966.37 (4) Å^3^	Prism, colorless
*Z* = 4	0.30 × 0.20 × 0.10 mm

### Data collection

**Table:**

Bruker SMART APEX diffractometer	986 reflections with *I* > 2σ(*I*)
Radiation source: fine-focus sealed tube	*R*_int_ = 0.039
Monochromator: graphite	θ_max_ = 27.5º
*T* = 100(2) K	θ_min_ = 2.6º
ω scans	*h* = −8→9
Absorption correction: None	*k* = −12→12
7349 measured reflections	*l* = −12→18
1164 independent reflections	

### Refinement

**Table:**

Refinement on *F*^2^	Secondary atom site location: difference Fourier map
Least-squares matrix: full	Hydrogen site location: inferred from neighbouring sites
*R*[*F*^2^ > 2σ(*F*^2^)] = 0.059	H-atom parameters constrained
*wR*(*F*^2^) = 0.206	*w* = 1/[σ^2^(*F*_o_^2^) + (0.1433*P*)^2^ + 0.309*P*] where *P* = (*F*_o_^2^ + 2*F*_c_^2^)/3
*S* = 1.11	(Δ/σ)_max_ = 0.001
1164 reflections	Δρ_max_ = 0.50 e Å^−^^3^
77 parameters	Δρ_min_ = −0.50 e Å^−^^3^
Primary atom site location: structure-invariant direct methods	

### Fractional atomic coordinates and isotropic or equivalent isotropic displacement parameters (Å^2^)

**Table:**

	*x*	*y*	*z*	*U*_iso_*/*U*_eq_	Occ. (<1)
O1	0.2012 (2)	0.00647 (14)	0.60172 (9)	0.0229 (5)	
O2	0.3277 (3)	0.2500	0.33669 (12)	0.0161 (5)	
N1	0.2007 (4)	0.2500	0.57847 (14)	0.0167 (6)	
C1	0.2372 (3)	0.17575 (18)	0.89087 (12)	0.0176 (5)	
H1	0.2434	0.1260	0.9475	0.021*	
C2	0.2281 (3)	0.09866 (19)	0.80904 (12)	0.0168 (5)	
H2	0.2280	−0.0026	0.8088	0.020*	
C3	0.2194 (3)	0.17598 (18)	0.72848 (11)	0.0144 (5)	
C4	0.2072 (3)	0.12658 (18)	0.63197 (13)	0.0171 (5)	
C5	0.1763 (4)	0.2500	0.47961 (16)	0.0176 (6)	
H5A	0.1034	0.3354	0.4610	0.021*	0.50
H5B	0.1034	0.1646	0.4610	0.021*	0.50
C6	0.3665 (4)	0.2500	0.43155 (16)	0.0175 (6)	
H6A	0.4404	0.3357	0.4486	0.021*	0.50
H6B	0.4404	0.1643	0.4486	0.021*	0.50
C7	0.4974 (4)	0.2500	0.28287 (17)	0.0210 (7)	
H7A	0.4642	0.2500	0.2177	0.031*	
H7B	0.5721	0.1647	0.2970	0.031*	0.50
H7C	0.5721	0.3353	0.2970	0.031*	0.50

### Atomic displacement parameters (Å^2^)

**Table:**

	*U*^11^	*U*^22^	*U*^33^	*U*^12^	*U*^13^	*U*^23^
O1	0.0377 (10)	0.0142 (8)	0.0166 (8)	0.0009 (6)	−0.0011 (6)	−0.0037 (5)
O2	0.0210 (11)	0.0178 (9)	0.0093 (9)	0.000	0.0003 (7)	0.000
N1	0.0282 (14)	0.0143 (11)	0.0075 (10)	0.000	0.0000 (8)	0.000
C1	0.0240 (10)	0.0185 (10)	0.0102 (9)	0.0008 (7)	0.0016 (6)	0.0018 (6)
C2	0.0237 (11)	0.0134 (8)	0.0133 (9)	−0.0006 (7)	0.0001 (7)	0.0017 (6)
C3	0.0189 (10)	0.0142 (9)	0.0101 (9)	0.0001 (7)	0.0003 (6)	−0.0012 (6)
C4	0.0258 (11)	0.0133 (9)	0.0121 (9)	0.0011 (7)	0.0006 (7)	0.0004 (6)
C5	0.0220 (14)	0.0216 (12)	0.0091 (12)	0.000	−0.0024 (9)	0.000
C6	0.0239 (15)	0.0197 (11)	0.0088 (12)	0.000	−0.0011 (9)	0.000
C7	0.0284 (17)	0.0196 (12)	0.0149 (12)	0.000	0.0043 (11)	0.000

### Geometric parameters (Å, °)

**Table:**

O1—C4	1.211 (2)	C3—C3^i^	1.389 (3)
O2—C6	1.412 (3)	C3—C4	1.486 (2)
O2—C7	1.432 (3)	C5—C6	1.514 (4)
N1—C4	1.398 (2)	C5—H5A	0.9900
N1—C4^i^	1.398 (2)	C5—H5B	0.9900
N1—C5	1.454 (3)	C6—H6A	0.9900
C1—C1^i^	1.394 (3)	C6—H6B	0.9900
C1—C2	1.398 (2)	C7—H7A	0.9800
C1—H1	0.9500	C7—H7B	0.9800
C2—C3	1.384 (2)	C7—H7C	0.9800
C2—H2	0.9500		
			
C6—O2—C7	112.1 (2)	N1—C5—H5A	109.5
C4—N1—C4^i^	111.9 (2)	C6—C5—H5A	109.5
C4—N1—C5	123.97 (11)	N1—C5—H5B	109.5
C4^i^—N1—C5	123.97 (11)	C6—C5—H5B	109.5
C1^i^—C1—C2	121.16 (10)	H5A—C5—H5B	108.1
C1^i^—C1—H1	119.4	O2—C6—C5	106.5 (2)
C2—C1—H1	119.4	O2—C6—H6A	110.4
C3—C2—C1	117.21 (17)	C5—C6—H6A	110.4
C3—C2—H2	121.4	O2—C6—H6B	110.4
C1—C2—H2	121.4	C5—C6—H6B	110.4
C2—C3—C3^i^	121.63 (11)	H6A—C6—H6B	108.6
C2—C3—C4	130.19 (16)	O2—C7—H7A	109.5
C3^i^—C3—C4	108.18 (9)	O2—C7—H7B	109.5
O1—C4—N1	124.48 (17)	H7A—C7—H7B	109.5
O1—C4—C3	129.64 (16)	O2—C7—H7C	109.5
N1—C4—C3	105.87 (15)	H7A—C7—H7C	109.5
N1—C5—C6	110.8 (2)	H7B—C7—H7C	109.5
			
C1^i^—C1—C2—C3	0.1 (2)	C3^i^—C3—C4—O1	−179.02 (19)
C1—C2—C3—C3^i^	−0.1 (2)	C2—C3—C4—N1	179.5 (2)
C1—C2—C3—C4	−179.47 (19)	C3^i^—C3—C4—N1	0.07 (17)
C4^i^—N1—C4—O1	179.03 (13)	C4—N1—C5—C6	−92.3 (2)
C5—N1—C4—O1	3.2 (4)	C4^i^—N1—C5—C6	92.3 (2)
C4^i^—N1—C4—C3	−0.1 (3)	C7—O2—C6—C5	180.0
C5—N1—C4—C3	−176.0 (2)	N1—C5—C6—O2	180.0
C2—C3—C4—O1	0.5 (4)		

Symmetry codes: (i) *x*, −*y*+1/2, *z*.
